# Rethinking body representations in autism across cultures

**DOI:** 10.3389/fpsyt.2025.1612219

**Published:** 2025-08-04

**Authors:** Joanna Mourad, Bernadette Grosjean, Nader Perroud, Katleen Bogaerts, Martin Desseilles, Bruno Bonnechère

**Affiliations:** ^1^ REVAL Rehabilitation Research Center, Faculty of Rehabilitation Sciences, Hasselt University, Diepenbeek, Belgium; ^2^ Technology-Supported and Data-Driven Rehabilitation, Data Sciences Institute, Hasselt University, Diepenbeek, Belgium; ^3^ Department of Psychology, University of Namur, Namur, Belgium; ^4^ Transition Institute, University of Namur, Namur, Belgium; ^5^ Retired, Los Angeles, CA, United States; ^6^ Department of Psychiatric Specialties, Department of Psychiatry, Geneva University Hospitals, Geneva, Switzerland; ^7^ Health Psychology, Faculty of Psychology and Educational Sciences, University of Leuven, Leuven, Belgium; ^8^ Department of PXL – Healthcare, PXL University of Applied Sciences and Arts, Hasselt, Belgium

**Keywords:** body representations, body image, body schema, culture, autism

## 1 Body representations

Body representations (BR) refer to dynamic neural multi-layered models that integrate sensory, motor, and cognitive processes, allowing individuals to perceive and interact with their bodies (1). Traditionally, BR have been divided into two primary components: body schema, which involves unconscious, action-based representations of the body, and body image, which pertains to conscious, perception-based understandings of the body (2). More recent frameworks extend this dichotomy proposing that BR are formed by a combination of real-time multisensory inputs and an internal “offline body model” that guides actions (3). Furthermore, the role of body memory includes unique representations of the body that develop during distinct developmental periods enabling to add a temporal depth to this system (4). In essence, BR are adaptable frameworks that are continuously updated through interactions with both the environment and culture, influencing how individuals perceive and navigate the world (5). These representations play a crucial role in sensory processing, self-awareness, motor coordination, and social interactions (6, 7).

In autism, BR manifest atypically, often contributing to sensory sensitivities, altered proprioception, and difficulties with motor skills (8, 9), impacting daily tasks like personal hygiene and meal preparation. Understanding these atypicalities requires tailored assessments. However, existing questionnaires and evaluations have primarily been developed in a limited set of developed nations, including France (10, 11), Saudi Arabia (12), Japan (13), the USA (14), the UK (15), Sweden (16) and Austria (17). This concentration in developed countries restricts the applicability of these tools. Despite cultural commonalities, biased assessments overlook global BR diversity, neglecting many cultures and hindering autism diagnosis and support worldwide. Consequently, future research must prioritize developing universally adaptable assessments that accommodate cultural and linguistic diversity, enhancing both accuracy and accessibility. Because cultural norms significantly shape how BR manifest and how autism is perceived, e.g., varying norms around eye contact and social engagement (18–20), it is essential to assess BR within these contexts. This necessity extends well beyond autism, as cultural beliefs and practices shape health-seeking behaviors and the interpretation of symptoms across different populations. Culturally grounded adaptations in autism interventions improve effectiveness, as emphasized by Keehn (21), and cultural beliefs influence care practices and treatment outcomes (21, 22). A one-size-fits-all approach to autism assessment may therefore overlook critical cultural nuances, potentially leading to misdiagnosis or ineffective interventions.

Therefore, this opinion highlights the importance of integrating cultural factors in the assessment of BRs in autism.

## 2 Defining the landscape of BRs research in ASD

Currently, the vast majority of the studies are being conducted in high-income countries (HIC), which leads to biased understandings of autism (23), limiting the generalizability of findings to non-Western populations. While 80% of the population lives in LMICs (23), research on autism in low- and middle income countries (LMICs) is still very limited, and many individuals remain undiagnosed due to the lack of culturally appropriate diagnostic tools (24–27). For example in China, diagnostic services are still developing, resulting in lower prevalence rates 39.23 per 10,000 compared to higher-income countries like the USA and Northern Europe (1% to 2.21%); this gap can be attributed to diagnostic tools that overlook cultural differences, language barriers, and varying levels of autism awareness (23–29). This disparity is evident in epidemiological studies, where 26 of 37 surveys were from high-income countries and only 11 from middle-income countries (average prevalence 0.47%), with no studies from low-income countries (18). In Nigeria and South Africa, researchers highlight persistent barriers such as low diagnostic awareness, cultural stigma, and a lack of regionally adapted screening tools (24, 25). Similarly, in India, Dey et al. conducted an online survey followed by an in-depth conversation with parents of autistic children and autistic adults emphasizing the need for culturally grounded autism priorities and tools (26). These studies demonstrate that underdiagnosis in LMICs is not solely due to economic constraints, but also to fundamental mismatches between Western-based diagnostic models and local sociocultural frameworks.

To better evaluate the research specifically performed around BR in ASD we performed a bibliometric analysis. Bibliometric analysis is a quantitative method for mapping scientific literature, allowing researchers to identify trends, collaboration networks, knowledge gaps and potential future research direction by analyzing publication metadata (30). Data for the present study was obtained from the WoS (accessed on 15 April 2025), using MeSH and title/abstract keywords related to autism spectrum disorder (e.g., “ASD,” “autism,” “Asperger’s syndrome”) and body representations (e.g., “body awareness,” “interoception,” “body schema,” “proprioception,” “sensorimotor integration,” “embodiment,” “motor imagery”). A total of 4,024 documents including research articles, review articles and book chapters formed the final dataset, with data processed using VOSviewer to visualize and map collaboration networks (31).

**Figure 1:**
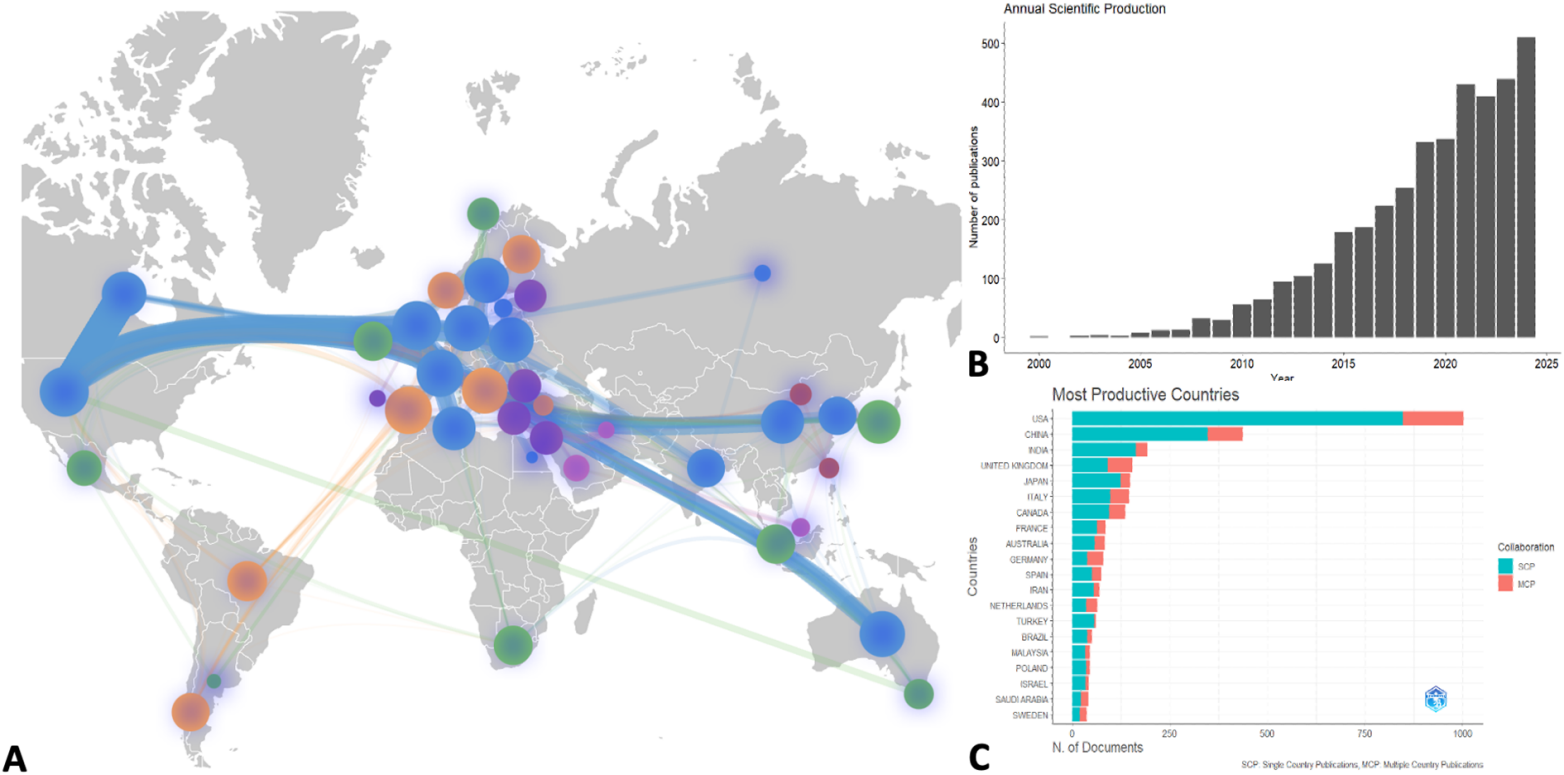
Bibliometric Analysis of studies on BRs in ASD. **(A)** Country collaboration map, the size of the bubble are proportional to the number of studies, the width of the connection is proportional to the number of collaborations, the color represents the different clusters with the most collaborations. **(B)** Annual scientific production; **(C)** Most productive countries, including single (green) and multiple country collaborations (red).

Before 2000 we observed that this concept was scarcely studied, but it has since experienced exponential growth. (**Figure 1B**). As for studies about the prevalence of ASD we observed that most, if not all the studies, are being performed in HIC (the 20 most significant contributor are presented in **Figure 1C**). Another important finding, potentially limiting the external validity of these studies, from a cultural point of view, is that most of the studies are being performed in one single country and that, as depicted in **Figure 1A**, there are almost no collaborations with LMICs.

## 3 The main challenges in assessing BRs in various cultures

### 3.1 Cultural background: between similarities and differences in regional patterns

One crucial aspect to consider is that cultural similarities across certain regions or countries can result in similar patterns in the manifestation of BR and other autism-related characteristics. Countries with shared cultural, historical, or linguistic backgrounds are more likely to exhibit comparable societal norms and attitudes towards body perception. This common cultural foundation can lead to similarities in research outcomes within these regions. For example, studies conducted in countries which share cultural and linguistic resemblances, often show analogous results in terms of BRs assessments and autism prevalence (23). Countries in the Middle East, like Saudi Arabia and Egypt, may exhibit parallel outcomes due to shared cultural and religious norms (32–36). Similarly, countries influenced by Latin European and Western biomedical traditions share cultural and scientific framework, public health and educational systems, ultimately leading to alike societal attitudes toward body perceptions. In Westernized cultures, particularly among women, dissatisfaction with body weight and appearance is common and has been linked to eating disorders, depression, and anxiety (19, 37). In contrast, other cultures may emphasize different physical ideals or interpret bodily differences, for instance overweight can be associated with the norm of beauty or even considered as healthy and wealth (18, 19, 38). Western diagnostic tools, such as those based on body image dissatisfaction, may not be applicable or relevant in non-Western contexts. Moreover, behaviors seen as socially deviant in one culture may be normalized or even valued in another.

While cultural norms are acknowledged to shape body representation (BR) (19, 39) the mechanisms by which these influences operate, particularly in autism, deserve a deeper exploration. Cultural environments structure early sensorimotor experiences (40, 41), including norms around physical touch, posture, bodily autonomy, and social body use. In autistic individuals, who often show atypical sensory processing, including altered proprioception, interoception, and tactile perception (42–44), culturally mediated sensory input may exert amplified or divergent developmental effects. For example, studies have shown that self-representation and multisensory integration are modulated by cultural experiences through plasticity in regions such as the insula and temporoparietal junction (45, 46), areas that are also implicated in BR and are known to function atypically in autistic individuals (47). Taken together, these findings suggest that culturally specific experiences dynamically shape BR neurodevelopment by influencing multisensory integration and bodily self-awareness, particularly in neurodivergent populations. Therefore, a culturally sensitive approach is critical for accurate assessment in autistic individuals. It is also important to consider the nature-nurture balance in developmental disorders. This distinction underscores the need to tailor assessment tools not only to regional cultural contexts but also to the underlying nature *vs* nurture profile of the condition being evaluated. Acknowledging cultural similarities and differences can thus facilitate the development of more accurate and universally adaptable diagnostic tools (35, 48, 49).

### 3.2 Language and communication

Language and communication barriers can also play a significant role in assessing BR, especially in multilingual and culturally diverse contexts (50). Body language and social communication, both shaped by cultural and linguistic norms, can present unique challenges for autistic individuals (51). Cultural variations in non-verbal communication may further complicate the interpretation of autistic individuals’ physical expressions. For example, while typically developing children tend to display open, outward-directed movements, autistic children often exhibit inward, closed postures and lighter movements. However, these differences should not be seen merely as deficits but rather as intrinsic aspects of their identity. As Gowen and collaborators suggest, it is no longer “one big clumsy mess” but “a fundamental part of [their] character” (52). Future research should explore how cultural expectations around body language influence the perception and interpretation of autistic individuals’ movements, rather than treating these differences as barriers.

## 4 A more culturally sensitive healthcare model

Several culturally sensitive models have been developed to address these issues and enhance patient-centered care (53). In response to increasing diversity, healthcare professionals, and policymakers are developing culturally competent services that align communication and care with patients’ values (54). For example, the Patient-Centered Culturally Sensitive Health Care Model has demonstrated that when healthcare providers exhibit cultural sensitivity, patient adherence to treatment improves among ethnically diverse patients, particularly low-income African American and non-Hispanic White Americans, seen in community-based primary care clinics. However, a recent scoping review highlights significant heterogeneity in cultural competence models and interventions, with most focusing on racial and ethnic diversity, particularly in minority populations, overlooking cultural diversity. This underscores the need for more inclusive models suited to Europe’s superdiverse populations and beyond (55).

Another crucial initiative is the ACT Cultural Model, which provides guiding principles for culturally competent care, emphasizing the integration of cultural sensitivity training into both undergraduate and postgraduate healthcare education. A meta-ethnographic review further underscores the importance of self-awareness, cultural sensitivity, and effective collaboration with patients and families (56, 57). Keehn et al. (21) applied the Ecological Validity Framework in Kenya to co-develop a caregiver training program, emphasizing the importance of community partnership and culturally grounded design. Their approach illustrates how interventions can be meaningful and scalable when co-created with local stakeholders. Additionally, Kang-Yi et al. (22) examined how community-level cultural beliefs profoundly shape both caregiver help-seeking behaviors and professional diagnostic pathways. Despite the widespread recognition of cultural competence, research on its impact remains limited, particularly in specialized areas such as autism assessment and intervention (58). Moreover, the challenges associated with autism diagnosis in low-resource settings highlight the urgent need for culturally sensitive assessment tools. The absence of culturally appropriate diagnostic tools and a shortage of trained professionals can severely hinder autism diagnosis and treatment, especially in diverse cultural contexts (59). Similarly, it has been shown that screening tools for autism are often unavailable or poorly understood in certain cultural settings, resulting in delays in diagnosis and intervention (60). Note that these issues are not unique to autism; they extend to other mental and physical health conditions, where cultural factors significantly impact the accessibility and effectiveness of diagnostic tools and treatments, this further highlights the absolute necessity of developing more culturally adapted and less language dependent assessment tools.

Beyond diagnosis, the experiences of individuals with autism and their families further illustrate the critical role of cultural factors in shaping both clinical outcomes and lived experiences. For instance, research highlights how social policies and cultural narratives influence the identities of individuals with autism, underscoring the need for policies that reduce stigma and promote understanding (61). Cultural perceptions do not only affect the diagnostic process but also shape societal attitudes, access to support, and the well-being of autistic individuals and their families. These challenges parallel those faced by individuals with other health conditions, where cultural stigma may impact treatment adherence and patient outcomes. **Table 1** summarizes key cultural influences on autism and outlines their implications for patients and the delivery of healthcare.

**Table 1:** Key aspects of autism that can be influenced by culture and language, highlighting the complexities involved in providing culturally sensitive care for autistic individuals across diverse global contexts.

Dimension	Description	Implications for the patients	Implications for healthcare providers
Cultural perception of autism	Varying cultural norms and beliefs about autism symptoms and behaviors	May lead to misdiagnosis or delayed diagnosis; affects social acceptance and support	Need to develop culturally sensitive diagnostic tools and interventions
Language barriers	Difficulty in expressing concerns or understanding diagnoses due to language differences	Poorer healthcare outcomes; challenges in accessing appropriate care	Requires use of interpreters or multilingual staff; need for translated materials
Body language and non-verbal communication	Cultural variations in interpreting body language and gestures	Autistic individuals’ behaviors may be misinterpreted or overlooked	Need for awareness of cultural differences in non-verbal communication
Diagnostic tools	Most tools developed in Western, high-income countries	May not accurately assess autism in diverse cultural contexts	Need to adapt and validate tools for different cultural settings
Body representations	Cultural differences in body perception and ideals	May affect how autistic individuals perceive and interact with their bodies	Requires culturally appropriate assessments of body representations
Healthcare access	Varying levels of autism awareness and available services across cultures	Unequal access to diagnosis and treatment, especially in low- and middle-income countries	Need to develop strategies for improving autism care in diverse global contexts
Social norms and expectations	Different cultural expectations for social behavior and interaction	May impact how autism symptoms are perceived and managed	Requires understanding of local social norms when assessing and treating autism
Stigma and acceptance	Varying levels of stigma or acceptance of autism across cultures	Affects willingness to seek diagnosis and treatment; impacts quality of life	Need to address cultural stigma and promote autism acceptance in healthcare settings

## 5 Call for action: bridging cultural gaps in autism and BRs

It is imperative that we address the significant cultural and geographical gaps in our understanding of BR. Current research predominantly hails from developed countries, leading to skewed insights and culturally limited assessment tools. This Western-centric focus neglects the diverse manifestations of autism that occur across different global cultures, potentially resulting in misdiagnoses and ineffective interventions.

To improve this, more inclusive and adaptable diagnostic tools that reflect a multitude of cultural contexts need to be developed. Researchers and practitioners should prioritize collaborations between HICs and LMICs to ensure a broader, more comprehensive understanding of BRs and autism. However, such collaboration must consider the economic and infrastructural constraints in LMICs. Funding, staffing, and technological gaps remain key barriers. To address feasibility, we propose several cost-effective and scalable approaches. Mobile health (mHealth) tools, such as app-based screening and tablet assessments, can be delivered in local languages and administered by trained lay health workers. These tools can be integrated into existing community-based health programs and public health systems, such as those supported by the WHO’s Mental Health Gap Action Program (mhGAP). Participatory co-design involving local clinicians, families, and autistic individuals is essential to ensure cultural relevance and uptake. Open-access diagnostic protocols and public–private partnerships can help mitigate financial constraints and reduce dependency on expensive, proprietary Western tools.

Investing in local research within LMICs and fostering cross-cultural partnerships will pave the way for diagnostic advancements that are both accurate and accessible worldwide.

Furthermore, integrating cultural sensitivity into autism assessments will improve their relevance and effectiveness. This involves tailoring interventions to align with regional norms, language proficiencies, and communication styles, ensuring that all individuals receive equitable care and support.

Additionally, cross-cultural adaptation and validation of diagnostic tools are crucial to ensure they account for local languages, cultural norms, and ecological contexts. Emerging technology-based, unsupervised methods for longitudinal assessment, such as digital phenotyping, offer promising solutions, particularly in regions with limited access to healthcare professionals (62). These innovations could help integrate BR assessments into natural environments, making diagnosis and intervention more accessible and ecologic.

Taken together, these solutions represent feasible, scalable strategies to address Western-centric limitations and promote culturally informed autism diagnostics in diverse global settings.

## 6 Conclusion

In conclusion, integrating cultural factors into autism assessment is a broader necessity in healthcare, as cultural contexts shape the understanding, diagnosis, and treatment of various mental and physical health conditions. Failing to consider these factors presents ethical and practical challenges, particularly in evaluating body representations, a core psychomotor function linked to autonomy and quality of life.

Autism research has historically been Western-centric, overrepresenting white, cisgender, and educated individuals while underrepresenting those from diverse ethnic, cultural, religious, and socioeconomic backgrounds, particularly in LMICs. This representativity bias has distorted the global understanding of autism, leading to misdiagnosis and inadequate support.

To address these disparities, investing in culturally adapted diagnostic tools and digital phenotyping enabling unsupervised, longitudinal assessments is essential. Cross-cultural collaboration among researchers will ensure more accurate, inclusive, and context-sensitive autism assessments.
